# Supplementary material for: A role for the fornix in temporal sequence memory
Source: Eur J Neurosci. 2023 Feb 28;57(7):1141–60. doi: 10.1111/ejn.15940 (PMC10336598; doi:10.1111/ejn.15940)
Supplement: Supplementary file 1 — Table S1. Mean along‐tract, bilaterally averaged tract microstructure metrics. Table S2. PCA loadings. Figure S1. Relationships between individual tract microstructure measures and behavioural data. The colours indicate positive and negative correlations according to the key to the left. Pearson r values are shown, and the asterisks indicate significant (at alpha level = .05) correlations. FWE‐AD: Axial Diffusivity. FWE‐FA: Fractional Anisotropy. FWE‐MD: Mean Diffusivity. FWE‐RD: Radial Diffusivity (with Free Water Elimination). ILF: Inferior Longitudinal Fasciculus. PHC: Parahippocampal Cingulum. NDI: Neurite Density Index. ODI: Orientation Dispersion Index. [file EJN-57-1141-s001.docx]

**A Role for the Fornix in Temporal Sequence Memory**

Marie-Lucie Read, Katja Umla‐Runge, Andrew D. Lawrence, Alison G. Costigan, Liang-Tien Hsieh, Maxime Chamberland, Charan Ranganath and Kim S. Graham.

**Supplementary Materials**

|  | **Fornix** | | **ILF** | | **PHC** | |
| --- | --- | --- | --- | --- | --- | --- |
|  | **Group mean** | **SD** | **Group mean** | **SD** | **Group mean** | **SD** |
| **FWE-FA** | 0.40 | 0.01 | 0.44 | 0.02 | 0.35 | 0.03 |
| **FWE-MD** | 0.09 x10^-2^ | 0.03 x10^-3^ | 0.07 x10^-2^ | 0.01 x10^-3^ | 0.07 x10^-2^ | 0.01 x10^-3^ |
| **FWE-RD** | 0.07 x10^-2^ | 0.03 x10^-3^ | 0.05 x10^-2^ | 0.02 x10^-3^ | 0.06 x10^-2^ | 0.02 x10^-3^ |
| **FWE-AD** | 0.14 x10^-2^ | 0.05 x10^-3^ | 0.11 x10^-2^ | 0.03 x10^-3^ | 0.10 x10^-2^ | 0.02 x10^-3^ |
| **NDI** | 0.45 | 0.03 | 0.51 | 0.03 | 0.46 | 0.02 |
| **ODI** | 0.15 | 0.01 | 0.19 | 0.02 | 0.24 | 0.02 |
| **PC1** | -2.60 | 0.42 | 1.30 | 0.38 | 1.30 | 0.32 |
| **PC2** | -0.04 | 0.53 | -1.43 | 0.87 | 1.47 | 0.97 |

**Table S1. Mean along-tract, bilaterally averaged tract microstructure metrics.**

|  | **PC1** | **PC2** |
| --- | --- | --- |
| **FWE-FA** | -0.027 | -0.674 |
| **FWE-MD** | -0.527 | 0.046 |
| **FWE-AD** | -0.521 | -0.126 |
| **FWE-RD** | -0.495 | 0.222 |
| **NDI** | 0.255 | -0.502 |
| **ODI** | 0.375 | 0.476 |

**Table S2. PCA loadings.**


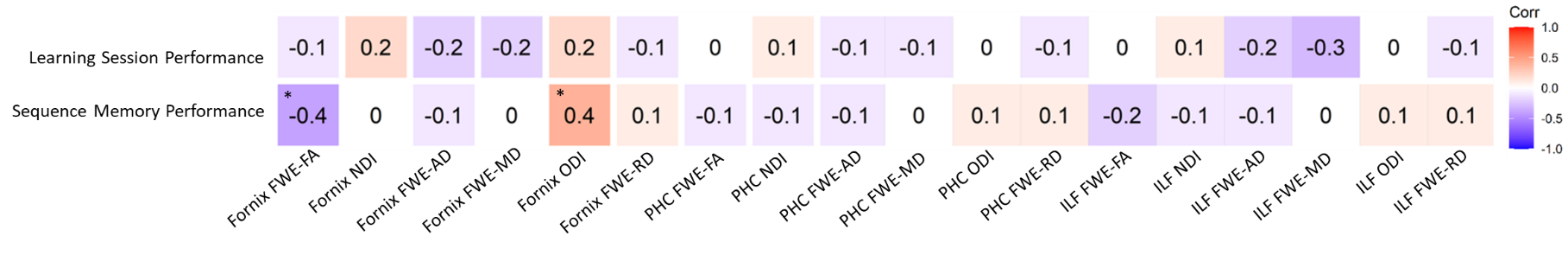
**Figure S1. Relationships between individual tract microstructure measures and behavioural data.**The colours indicate positive and negative correlations according to the key to the left. Pearson r values are shown, and the asterisks indicate significant (at alpha level = 0.05) correlations.
FWE-AD: Axial Diffusivity. FWE-FA: Fractional Anisotropy. FWE-MD: Mean Diffusivity. FWE-RD: Radial Diffusivity (with Free Water Elimination). ILF: Inferior Longitudinal Fasciculus. PHC: Parahippocampal Cingulum. NDI: Neurite Density Index. ODI: Orientation Dispersion Index.
